# Mathematics anxiety and number processing: The link between executive functions, cardinality, and ordinality

**DOI:** 10.1177/17470218241234041

**Published:** 2024-02-27

**Authors:** Kenny Skagerlund, Mikael Skagenholt, Ulf Träff

**Affiliations:** 1 Department of Behavioural Sciences and Learning, Linköping University, Linköping, Sweden; 2 JEDILab, Division of Economics, Department of Management and Engineering, Linköping University, Linköping, Sweden; 3 Center for Social and Affective Neuroscience, Department of Clinical and Experimental Medicine, Linköping University, Linköping, Sweden

**Keywords:** Mathematics anxiety, number sense, ordinality processing, number processing, executive functions

## Abstract

One important factor that hampers children’s learning of mathematics is math anxiety (MA). Still, the mechanisms by which MA affects performance remain debated. The current study investigated the relationship between MA, basic number processing abilities (i.e., cardinality and ordinality processing), and executive functions in school children enrolled in grades 4–7 (*N* = 127). Children were divided into a high math anxiety group (*N* *=* 29) and a low math anxiety group (*N* = 31) based on the lowest quartile and the highest quartile. Using a series of analyses of variances, we find that highly math-anxious students do not perform worse on cardinality processing tasks (i.e., digit comparison and non-symbolic number sense), but that they perform worse on numerical and non-numerical ordinality processing tasks. We demonstrate that children with high MA show poorer performance on a specific aspect of executive functions—shifting ability. Our models indicate that shifting ability is tied to performance on both the numerical and non-numerical ordinality processing tasks. A central factor seems to be the involvement of executive processes during ordinality judgements, and executive functions may constitute the driving force behind these delays in numerical competence in math-anxious children.

## Introduction

Proficiency in mathematics is arguably one of the most important skills that we aim to foster when sending our children to elementary school. The negative implications for those unfortunate enough to attain an inadequate level of competence cannot be overstated, in terms of academic success (Butterworth, 2010), professional success (Paulos, 1988), and overall economic and psychological well-being (Parsons & Bynner, 2005). It is therefore understandable, and indeed welcome, that many researchers from a wide variety of disciplines try to unveil and identify what factors ultimately determine how individuals successfully learn mathematics. From a psychological perspective, significant headway has been made in the last couple of decades in terms of isolating cognitive factors that underlie and scaffold mathematical learning, such as logical reasoning (e.g., de Jong & van der Leij, 1999; Geary, 2013), working memory capacity (e.g., Bull et al., 2008; Szűcs et al., 2014), and executive functions (e.g., Geary, 2010; Lefevre et al., 2013; Szűcs et al., 2014). In addition, extensive research has established that domain-specific number processing abilities are predictive of children’s learning of mathematics, such as the ability to estimate and manipulate non-symbolic quantities (i.e., *number sense;* Decarli et al., 2023) and decode and understand the meaning of number symbols (i.e., Arabic digits; Merkley & Ansari, 2016).

Mathematical learning is of course not solely dependent on purely cognitive processes, but also on social and emotional factors. One such important emotional factor that has received increasing attention in the literature is mathematics anxiety (MA), which can be defined as “. . .feelings of tension and anxiety that interfere with the manipulation of numbers and the solving of mathematical problems in a wide variety of ordinary life and academic situations.” (Richardson & Suinn, 1972, p. 551). Previous research has shown that MA is inversely correlated with math achievement (Barroso et al., 2021; Caviola et al., 2021; Ma, 1999) and is distinct from other forms of anxiety, such as test anxiety and trait anxiety (Hembree, 1990; Kazelskis et al., 2000), and its prevalence estimates range from 33% in 15-year-olds (Organization for Economic Co-Operation and Development, 2013), 25% in college students (Chang & Beilock, 2016), and 17% in the general population (Ashcraft & Moore, 2009). In children, the prevalence rate is dependent on age, and studies suggest that the level of MA in younger children in elementary school is quite low (Szczygiel & Pieronkiewicz, 2021) but apparent as early as first grade (Kucian et al., 2018; Ma, 1999; Ramirez et al., 2013). Importantly, the level of MA in children substantially increases with age throughout elementary school and peaks in young adulthood (Dowker et al., 2012, 2016; Kucian et al., 2018). Therefore, it is of utmost importance that we understand how MA develops, its origins, and the mechanisms by which it interferes with math learning.

As will be evident in the literature review below, several suggestions have been made as to what cognitive abilities or numerical skills may underlie and fuel the development of MA, and there are conflicting findings depending on what measures are used and whether children or adults are the target population (Dowker et al., 2016). There is also an ongoing debate as to the causal direction of poor math performance and MA. Poor math ability and experiences may result in increased anxiety (i.e., the Deficit Theory). It has also been suggested that the causal direction is reversed, such that feelings of anxiousness about math reduce performance (i.e., the Debilitating Anxiety Model). Recent research suggests that these effects may act in tandem in a bidirectional relationship over time (Carey et al., 2016). The purpose of the current study was to focus on disentangling these competing accounts in middle school children, which is arguably the most important and understudied population when considering that potential interventions and resources should be targeted as early as possible in development.

Although significant progress has been made in terms of teasing out exactly how MA interferes with mathematical learning, several competing hypotheses have been proposed and it is still unclear the degree to which they may interact in concert. One clear link may be attributed to avoidance behaviour due to initial struggles and failures in mathematics, thus limiting one’s engagement with material and concepts that are pertinent to an individual’s progress and understanding of the subject matter (Ashcraft, 2002; Douglas & Lefevre, 2017; Hembree, 1990). Children with pronounced and specific difficulties in learning mathematics (i.e., developmental dyscalculia; DD) often express negative emotions towards mathematics, and research has indicated that children with DD are twice as likely as typical achievers to express high levels of MA (Devine et al., 2018). However, the authors also conclude that high MA is not exclusively linked to poor math performance, given that the majority of children with high MA show typical math performance or above. One prominent account points to the likelihood that experience of negative emotions and stress reduces working memory resources available for mathematical problem solving, and ample support for this account has been put forth (e.g., Ashcraft & Faust, 1994; Ashcraft & Kirk, 2001; Skagerlund et al., 2019). The importance of WM resources in carrying out mathematical operations, and ultimately to overall mathematical achievement, and to MA is well-documented, but a related body of evidence also supports the notion that other aspects of executive functions (EF) may be implicated as well. The role of EF and its link to math anxiety (MA) is often discussed using the Attentional Control Theory (ACT), developed by Eysenck et al. (2007), as a backdrop. Here, anxiety acts as a disrupter of individuals’ ability to control attentional processes and impairs efficiency. EF is an umbrella term for different cognitive control processes that are involved in the regulation of cognition and behaviour (Miyake et al., 2000; Miyake & Friedman, 2012) and thus relates to attentional processes.

Conceptualisations of EF vary, but a prominent model has been advocated by Diamond (2013) and Miyake and Friedman (2012), where EF is divided into three main components: working memory (updating), shifting, and inhibition. These three components interact during cognitive processing and are subserved by a central executive network in the brain, comprising nodes in the dorsolateral prefrontal cortex and the posterior parietal cortex (Menon & Uddin, 2010; Sherman et al., 2014).

A study by Pizzie and colleagues (2020) found that both WM and shifting were impacted in high-math-anxious university students, where they could also identify lower neural activity in the central executive network, highly involved in arithmetical processing, comprised of areas such as the bilateral intraparietal sulcus (IPS) and lateral prefrontal cortex. Corroborating evidence for the role of EF in MA can also be found in a recent study in middle school children, where Zivkovic et al. (2022) demonstrated that the shifting component of EF specifically was implicated in children experiencing MA. A recent study by Li et al. (2023) used eye-tracking to investigate potential deficits in attentional control during math problem solving in math-anxious elementary and middle school students. The authors found, consistent with ACT, that students with higher MA seemed to fail to disregard task-irrelevant distractors during problem solving, which would be consistent with the notion that aspects of EF are involved in the relationship between MA and math performance deficits (Li et al., 2023).

Other researchers have suggested that MA develops because of difficulties with processing basic numerical information, where one such example comes from a study by Maloney et al. (2011), who found that undergraduate students with higher MA performed worse on a simple digit comparison task. A similar account also points to deficits in basic number processing, but instead highlights the role of the innate approximate number system (ANS) as the culprit behind the struggles in handling numerical information, in turn resulting in MA (Lindskog et al., 2017; Maldonado Mosco et al., 2022; Maloney et al., 2011; Núñez-Peña & Suárez-Pellicioni, 2014). Yet another, more recent account maintains that individuals with high MA show poorer performance on numerical ordinality tasks (Colomé & Núñez-Peña, 2021). Ordinality is an important characteristic of how children and adults understand numbers and refers to the fact that numbers not only convey cardinal information but also that they are embedded ordinally in a numerical sequence along a number line (Lyons & Beilock, 2011; Sasanguie et al., 2017; Sasanguie & Vos, 2018). Children’s understanding of numbers involves both cardinal information and being able to recite the count list (“one, two, three. . .”), which is learned early in childhood, but a more sophisticated understanding of how numbers can be arranged in an ordinal sequence with varying distances develops in later school years (Hutchison et al., 2022). Mounting evidence suggests that an affinity with ordinality processing is a stronger predictor of mathematical skills than number processing tapping into pure cardinality processing (Lyons et al., 2014) in 10- to 12-year-olds, but little is known whether ordinality processing could be tied to MA in grade school. The majority of studies that have found links between MA and numerical processing skills, such as cardinal characteristics measured using digit comparisons tasks or tasks measuring ANS acuity, have been established in young adults (e.g., Dietrich et al., 2015; Douglas & Lefevre, 2017; Georges et al., 2016; Lindskog et al., 2017; Maloney et al., 2011; Núñez-Peña & Suárez-Pellicioni, 2014; Pletzer et al., 2015). Studies in middle school children with MA often report differences in WM or EF (e.g., Justicia-Galiano et al., 2017; Mammarella et al., 2015; Pizzie et al., 2020; Zivkovic et al., 2022).

It is possible that the developmental trajectory of MA is differentially dependent on various cognitive abilities and numerical abilities that, in interaction with social and environmental factors, ultimately drive this development. One potential explanation could be that domain-general cognitive abilities, such as EF (Bull & Lee, 2014), are tightly linked to mathematical abilities and subsequent manifestation of MA in elementary school, with the consequence that these children and adolescents are less inclined to engage with mathematics curricula and subject matter throughout their educational careers. This, in turn, may affect the degree to which they hone their magnitude processing skills, non-symbolically or symbolically, compared with their less anxious peers, which results in poorer performance on basic number processing tasks apparent in studies on young adults. Nevertheless, one recurring finding is that ordinality processing in middle school children is a strong predictor of mathematical ability, more so than cardinality (e.g., symbolic number comparison or non-symbolic approximate number comparison tasks; Lyons et al., 2014). It is so far unknown whether MA can be linked to ordinality processing. Given that ordinality processing was affected in adults with MA (Colomé & Núñez-Peña, 2021), this question is ripe for investigation. Interestingly, it has also been argued that WM and EF are critical components involved in making these ordinal decisions (Lyons & Beilock, 2009) insofar as demands on WM and attention increase when judging unordered trials as being correct/incorrect, in contrast to ordered trials (e.g., “1 2 3”) that are readily available in long-term memory as children are more exposed to instances of correctly ordered sequences in the count list.

The objective of the current study was to administer a comprehensive battery of tests, including domain-general cognitive abilities (e.g., WM and EF) and number processing skills (including cardinal and ordinal number processing skills) in middle school children deemed to have high MA and to children with low MA to see the link between these abilities and how they relate to MA. To get a more thorough understanding of children’s ordinality skills, we also administered a non-numerical ordinal sequencing task, to see whether children with MA have a more general difficulty with ordering elements in a sequence, or whether it is circumscribed to the numerical domain alone.

Working memory is believed to provide a flexible and efficient mental workspace that is involved in handling the storage and updating of task-relevant information involved in complex arithmetic tasks (Fuchs et al., 2010; Geary, 1993). Working memory ability has been associated with mathematical ability in early childhood, and studies on children with DD often report—although often inconsistently—lower working memory abilities in this population (e.g., Mammarella et al., 2018; Szűcs et al., 2013). These findings may depend on which aspect of WM is measured (e.g., verbal WM or visuospatial WM). Interestingly, one study found that children with developmental dyscalculia struggle with serial order WM but not necessarily with WM capacity in general, such that being able to correctly order elements in WM is a characteristic of learning disabilities in mathematics (Attout & Majerus, 2015). Therefore, we expect that the inclusion of a non-numerical ordinality task will yield additional information on whether MA could be tied to a more general ordinal processing skill, irrespective of the type of information.

### The present study

The main goal of the current study was to try to disentangle the mechanisms by which MA may interfere with mathematical abilities in children, where we focus on aspects of EF and ordinality processing as primary targets of the investigation. Data were collected from a sample of children enrolled in grades 4–7 (*N* = 127) in middle school. Based on this sample, children were then divided into two groups in the upper and lower quartiles of their rated MA, where the group with the highest rated MA was judged to display “high” MA, and the group in the lower end of the distribution were judged to have “low” MA. This allows us to address the following questions:

1. Are children with high MA characterised by lower EF resources?
2. Do children with high MA display poorer performance on basic number processing tasks?

### Hypotheses

Based on previous research, we formulated the following main hypotheses:

(i)  Children with high MA will show a lower working memory span on a verbal working memory task than children with low MA.
(ii)  Children with high MA will show slower performance on a shifting task (measuring one component of EF) than children with low MA.
(iii) Children with high MA will show poorer performance on a numerical ordering task (i.e., ordinality) than children with low MA.
(iv) Children with high MA will show poorer performance on a non-numerical ordering task (i.e., ordinality) than children with low MA.

## Method

### Participants

Children were recruited from participating middle schools in southern Sweden and were enrolled in grades 4–7. The sample (*N* = 127; 4th grade = 13, 5th = 26; 6th = 48; 7th = 40) consisted of 63 boys and 64 girls with a mean age of 12.64 (*SD* = 1.04, range = 10.25–14.75). All children had Swedish as their native language and had normal or corrected-to-normal vision. Parents gave informed consent for their children to participate. The study was approved by the local ethics committee and conformed to the Helsinki Convention.

### Assessment of mathematical ability

#### Arithmetic calculation

This test consisted of eight addition and eight subtraction problems written in Arabic numerals that became progressively more difficult (e.g., 56 + 47; 545 + 96; 824–488; 11,305–5,786). Each problem was presented horizontally (e.g., 545 + 96), with each subsequent problem separated vertically on a separate line. The children had to solve as many problems as possible within 10 min, and they only had paper and pencil at their disposal. The total number of problems to solve was 16, and each correctly solved problem yielded 1 point.

#### Arithmetic fluency

This task targeted arithmetic fluency and was divided into three subtasks for addition, subtraction, and multiplication. Children were presented with 81 arithmetic problems for the addition and subtraction subtasks and 58 problems for the multiplication subtask and were allowed 1 min to solve as many as possible. The tasks were completed by providing written answers on a piece of paper (e.g., 1 + 6 = 7). The children were instructed to solve as many problems as possible within the time limit. Scores were calculated both as sums of correct answers for each arithmetic operation and by combining the total number of correct answers across all three subtasks.

### Assessment of general cognitive abilities

#### Fluid intelligence

Raven’s (1976) Standard Progressive Matrices was administered as a measure of fluid intelligence. Only Sets B, C, and D were used to measure fluid intelligence, due to the overall mental demands of the entire test battery. This approach has been used successfully before (e.g., Skagerlund & Träff, 2014). The raw scores were used as the dependent variable.

#### EF

Verbal working memory was measured using a word recall task (cf. Östergren & Träff, 2013). Participants were presented with a list of words, read aloud by the experimenter, ranging from a span of two to seven words. Following each word, the participant was asked to determine if the word was an animal (e.g., by answering “yes” for dog and “no” for bus), after which the experimenter read the next word. After presenting the list of words, participants were instructed to recite the two words in the correct serial order. Two trials were given at each span size. The participant had to recall the words in the correct serial order for at least one of the two trials to advance to the next span size. Testing continued until the child failed to correctly recall the words of both trials of one span size. Each correctly recalled word yielded one point, and the total number of correctly recalled words (in absolute order) across all lists was used as the dependent variable.

Shifting ability was measured using the trail-making test (van der Sluis et al., 2004) in a paper-and-pencil format and contained two conditions. The first condition (A) contained 22 circles, each containing a digit, whereas the second condition (B) also contained 22 circles with either a digit or a letter. In condition A, the task was to draw a line and connect the circles in ascending order as quickly as possible. In condition B, the children are instructed to draw the line and connect the circles in ascending and alternating order (1-A-2-B-3C, etc.) as quickly as possible. The dependent measure was the number of seconds for each condition it took for the participant to complete the task. The condition requiring shifting between digits and letters was used as an index of shifting ability.

### Assessment of number processing skills

#### Symbolic number discrimination

Participants were presented with 32 trials consisting of 2 single digits presented simultaneously next to each other on a computer screen (cf. Träff et al., 2017). Participants were instructed to select the numerically larger digit by pressing either the left (F) or right (J) button on a computer keyboard, corresponding to the left and right digit. Before each trial, a fixation cross was displayed for 1,000 ms, after which two digits were presented and remained exposed to the child until he or she pressed a button. All digit pairs were presented twice, with the second instance being a mirrored version of the first (e.g., 2 vs. 3 and 3 vs. 2). Two numerical distances were used for near-distance (i.e., 1) and far-distance (i.e., 4–5) trials. Response time were only calculated for correct responses, excluding response times below 200 ms or 2 *SD* above the average individual response time. The task was administered using SuperLab 4.5 (Cedrus Corporation, San Pedro, CA, USA).

#### Non-symbolic number discrimination

The non-symbolic number discrimination task was administered using the Panamath software (version 1.22; Halberda et al., 2008). Participants were presented with two dot arrays for each of the 106 trials (ranging from 8 to 26 blue or yellow dots), where the objective was to select the more numerous array using the corresponding right-side (F) or left-side (J) response keys. Each array was presented for 984 ms to prohibit enumeration or counting strategies. Four numerosity ratio bins were used (1.14–1.27, 1.27–1.41, 1.46–1.62, and 2.40–2.67). Half of all trials contained more blue than yellow dots. Surface area and dot size were controlled in half of the trials. Response time and accuracy measures were used to calculate participants’ ANS acuity (Weber fraction).

#### Ordinal sequence processing

Two separate computer-based tasks were used to measure numerical and alphabetical ordinal processing. In the numerical task, the children were presented with 61 triplets consisting of single digits (1–9). Thirty trials were presented in a correct ascending or descending order (e.g., 1-2-3, 7-5-3), while the remaining trials were in an incorrect mixed order (e.g., 1-5-3). Two numerical distances were used for near-distance (i.e., 1) and far-distance (i.e., 2–3) trials. Each trial remained visible on screen until participants registered their answers. The participants were instructed to determine whether the numerical sequence was “correct” or “incorrect” (mixed order) and answer using two different response keys (“J”-key for correct and “F”-key for incorrect). Response times were calculated for correct responses, excluding response times below 200 ms or 2 *SD* above the average individual response time and used as dependent variables.

In the alphabetical ordinal sequence task, the overall setup of the task was similar to the numerical ordering task. The participants were presented with 61 triplets consisting of alphabetical letters ranging from A to L. The participants were instructed to determine whether the triplet was presented in the correct ascending or descending order (e.g., A-B-C, H-F-D) or a mixed incorrect order (e.g., A-C-B) according to the alphabet. The same number of correct and incorrect items, as well as sequential distances, were used as previously described for the numerical task. As in the numerical ordinal task, response times and accuracies were calculated for correct responses only, and outlier responses (>2 *SD* above individual responses) and response times < 200 ms were discarded.

### Mathematics anxiety

To measure individual MA, we administered the Stanford Early Mathematics Anxiety Scale (SEMA; Wu et al., 2012). Although initially developed to target younger children (8-year-olds), the SEMA has been shown to exhibit good psychometric characteristics in older children as well (3rd to 6th grade; Sánchez-Pérez et al., 2021). The SEMA questionnaire contains 20 items, half of which assess how anxious students feel about different mathematical problem-solving situations (e.g., “Daisy has more money than Ernie. Ernie has more money than Francesca. Who has more money—Daisy or Francesca?”), while the other half concerns social and testing situations (e.g., “You are about to take a math test”). Each question was presented on a piece of paper while also read aloud by the experimenter, and after each question, the participants were asked to rate how anxious they felt. The ratings were made on a five-point Likert-type scale. As a visual aid, the participants also saw a piece of paper with five different smileys, each depicting different stages of anxiousness, from “Not nervous at all” (1) to “Very, very nervous” (5). The summed score on the SEMA was used as an index of MA.

### Procedure

The study was conducted over two sessions taking place in a secluded room at the participating schools, one group (two to four individuals) and one individual session, and both sessions were completed within 1 month. Instructions were read aloud from a printed manuscript by the experimenter, and all tests were administered in the same order for all participants. The order was fixed but designed in such a way as to take task difficulty, fatigue, and boredom into consideration to remain constant for all participants. Computer-based tasks were run on a laptop using SuperLab PRO 4.5. During the group session, the following tasks were administered: Raven’s Standard Matrices, arithmetical calculation, arithmetical fluency, and the SEMA questionnaire. The remaining tests (basic numerical skills and EF) were performed during the individual session.

## Results

### Analytic approach and group composition

Prior to data analysis of computerised tasks, intra-individual trials were examined to remove outliers; RTs < 200 ms were removed, as were RTs > 2 *SD* of the mean response time within a test. Table 1 provides an overview of all variables and their correlations, including means and standard deviations for all tasks. Based on the mean rating of the entire sample on the MA questionnaire (*M* = 35.41, *SD* *=* 10.73), we divided the sample into a high math anxiety group (HMA) and a low math anxiety group (LMA) based on the lowest quartile and the highest quartile (see Table 2 for an overview). The HMA group consisted of 29 children (MA score ⩾ 41, *M* = 50.84, *SD* *=* 7.90) and the LMA group consisted of 31 children (MA score ⩽ 27, *M* = 24.17, *SD* = 1.98). The two groups showed significant differences in MA scores after correcting for violation of the assumption of equal variances, *t*(34.01) *=* 18.19, *p* < .001, *d* *=* 4.56. There was also a significant difference in age between the groups, such that the HMA group was older than the LMA group, *t*(58) = 3.71, *p* < .001, *d* = 0.96. Given our multiple hypotheses and corresponding tests, we control familywise error by Bonferroni correcting (α/4) main effects such that α-level is set at .0125 for our main analyses.

**Table 1.** Descriptive statistics, and correlations among the tasks used in the study (*N* = 127). Significant correlations in bold (*p* < .05).

Tasks	*M*	*SD*	2	3	4	5	6	7	8	9	10	11	12	13	14
1. Mean age (years)	12.64	1.04	**−.27**	.11	**.36**	**.32**	**−.24**	−.06	**−.36**	−.17	−.01	−.15	**−.26**	**−.30**	**−.35**
2. Fluid intelligence (raw score)	27.83	4.82	—	.05	**−.43**	**−.21**	**.45**	**.26**	.01	.06	**−.23**	−.04	**.41**	.12	**.30**
3. Verbal working memory (hits)	22.54	7.42		—	−.11	−.11	.06	.17	.08	**.19**	.02	.10	**.24**	.08	.09
4. Executive function—shifting (s)	78.37	31.37			—	**.25**	**−.44**	**−.38**	−.06	−.12	.03	**.21**	**−.37**	−.01	**−.28**
5. Math Anxiety (raw scores)	35.43	10.69				—	**−.35**	**−.46**	.05	−.10	.04	**.21**	−.15	.04	−.12
6. Arithmetic calculation	8.32	2.78					—	**.53**	−.11	.06	**−.23**	**−.22**	**.33**	.00	**.24**
7. Arithmetic fluency (correct)	68.23	21.95						—	**−.33**	.10	**−.20**	**−.48**	.12	**−.22**	.09
8. Digit comparison—all (ms)	691	132							—	**.41**	**.25**	**.51**	**.21**	**.51**	**.30**
9. Digit comparison—all (Corr)	30.13	1.64								—	−.08	.14	**.27**	.15	**.26**
10. Non-symbolic number discr. (*w*)	.22	.43									—	−.01	**−.18**	−.10	**−.31**
11. Numerical ordinality—all (ms)	2,406	935										—	−.18	**.65**	**.23**
12. Numerical ordinality—all (Corr)	51.35	6.63											—	**.44**	**.62**
13. Alphabetical ordinality—all (ms)	4,043	1,721												—	**.58**
14. Alphabetical ordinality—all (Corr)	45.46	8.97													—

**Table 2.** Descriptive data (raw scores) for children in the HMA group and the LMA group.

	HMA		LMA	
	*M*	*SD*	*M*	*SD*
*N* (number of boys)	31 (12)		29 (18)	
Mean age (years)	13.00	0.92	12.06	1.02
Fluid intelligence (raw scores)	26.39	5.34	29.21	4.46
Verbal working memory (hits)	20.52	8.75	23.64	7.18
Executive function—shifting (s)	91.29	42.38	67.27	23.68
Math Anxiety (raw scores)	50.84	7.90	24.17	1.98
Arithmetic calculation	7.19	3.26	9.41	2.18
Arithmetic fluency (Correct)	54.00	20.96	78.93	18.24
Digit comparison—all (ms)	703	137	680	119
Digit comparison—all (Correct)	30.10	1.56	30.38	1.11
Digit comparison—Dist. 1 (ms)	749	150	719	127
Digit comparison—Dist. 1 (Correct)	14.29	1.21	14.14	0.99
Digit comparison—Dist. 4-5 (ms)	661	136	647	121
Digit comparison—Dist. 4-5 (Correct)	15.81	0.79	16.24	0.79
Non-symbolic number discr. (Weber fraction)	0.19	0.08	0.18	0.12

HMA = High Math Anxiety; LMA = Low Math Anxiety.

### Math abilities and MA

Initial analyses of potential differences in math abilities across MA groups showed that both fluency and arithmetic calculation scores are significantly different. The HMA group performed worse on fluency (*M* = 54.00, *SD* = 20.96), *t*(58) = −4.50, *p* < .001, *d* = −1.27, than the LMA group (*M* = 78.93, *SD* = 18.24). The HMA group also performed worse on arithmetic calculation (*M* = 7.19, *SD* = 3.26) than the LMA group (*M* = 9.41, *SD* = 2.18), *t*(58) = −3.08, *p* = .003, *d* = −0.80.

### Fluid intelligence, EF, and MA

**Figure 1.**
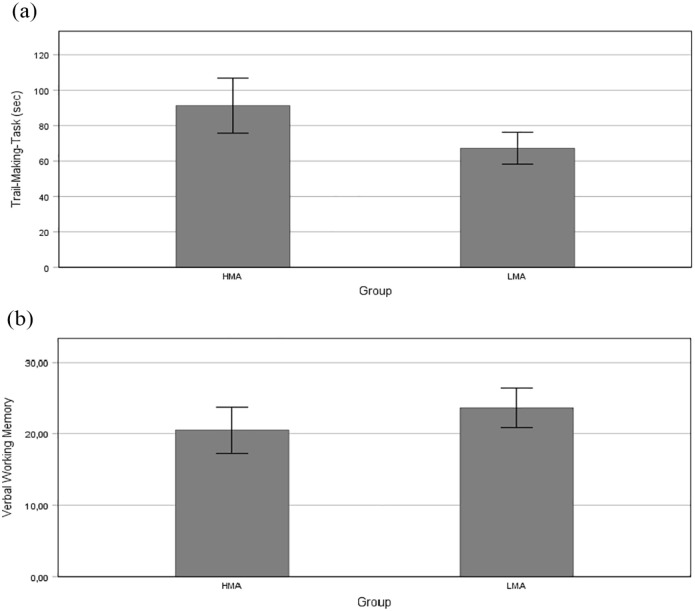
Overview of performance on the shifting and verbal working memory tasks. (a) Trail-making shift (shifting ability). (b) Verbal working memory.

In terms of fluid intelligence, there was a significant difference between the groups, where the LMA group (*M* = 29.21, *SD* = 4.46) scored higher than the HMA group (*M* = 26.39, *SD* = 5.34), *t*(58) = −2.21, *p* *=* .031, *d* *=* −0.57. Concerning verbal working memory, the HMA group (*M* = 20.52, *SD* = 8.75) did not show any difference in verbal working memory ability (*p* *=* .14), compared with the LMA group (*M* = 23.64, *SD* = 7.18). On the shifting task, however, the HMA group (*M* = 91.29, *SD* = 42.38) showed slower performance than the LMA group (*M* = 67.27, *SD* = 23.68), *t*(47.68) = 2.73, *p* *=* .009, *d* = 0.69. See Figure 1 for an overview.

### Cardinal number processing and MA

Comparison of the groups on non-symbolic number discrimination, measured using the individual Weber fractions as an index of ANS acuity, revealed no differences between the HMA group (*M* = .19, *SD* = .08) and the LMA group (*M* = .18, *SD* = .12), *t*(58) = 0.18, *p* = .855. Looking at the response times, there was no difference between the groups either (*t*(58) = 0.12, *p* = .908), with very similar response times between the HMA group (*M* = 953 ms, *SD* = 392 ms) and the LMA group (*M* = 942 ms, *SD* = 327 ms). To evaluate the performance on the symbolic number discrimination task, we performed a split-plot analysis of variance (ANOVA) with group as the between factor and distance as the within factor for both response times and accuracies. For response times, summarised in Table 2, there was a significant effect of distance, *F*(1,58) = 62.84, *p* < .001, partial η^2^ = .52. There was, however, no effect of group or interaction (*p*s > .436). For number of correct trials, again found in Table 2, there was an effect of distance, such that children committed more errors on the near-distance trials than on far-distance trials, *F*(1,58) = 105.00, *p* < .001, η^2^ = .64. Again, there was no effect of group nor interaction (*p*s > .102).

### Ordinal sequence processing and MA

To evaluate performance on the ordinal sequencing tasks (see Table 3 for an overview), we conducted separate analyses for the numerical task and the alphabetical task. In addition, separate analyses for trials in the correct order (i.e., half of all trials) and the total trials irrespective of whether they were in the correct order. This way, we could determine whether we could observe the reverse distance effect (RDE) on correct trials, meaning that participants are faster to respond to correct trials that are separated by one unit than to trials that are separated by two or three units in the count list and the alphabet. For these different analyses, we performed split-plot ANOVAs with group as between factor and distance as within subjects factor for both the number of correct trials and response times.

**Table 3.** Descriptive data (raw scores) for children in the HMA group and the LMA group ordinality tasks.

	HMA		LMA	
	*M*	*SD*	*M*	*SD*
Numerical ordinality—all (ms)	2,753	1,030	1,963	547
Numerical ordinality—all (Correct)	50.13	7.95	52.34	5.53
Numerical ordinality—Dist 1 (ms)	2,760	1,027	1,949	530
Numerical ordinality—Dist 1 (Correct)	24.42	3.11	24.20	2.41
Numerical ordinality—Dist 2–3 (ms)	2,750	1,109	1,969	595
Numerical ordinality—Dist 2–3 (Correct)	25.58	5.99	28.00	3.74
Numerical ordinality—ordered—Dist 1 (ms)	2,538	968	1,732	527
Numerical ordinality—ordered—Dist 1 (Correct)	12.58	1.29	12.76	1.15
Numerical ordinality—ordered—Dist 2–3 (ms)	2,708	1,388	1,927	571
Numerical ordinality—ordered—Dist 2–3 (Correct)	11.68	4.71	13.17	3.41
Alphabetic ordinality—all (ms)	4,388	1,850	3,777	1,462
Alphabetic ordinality—all (Correct)	45.23	8.88	46.71	8.46
Alphabetic ordinality—Dist 1 (ms)	4,334	1,755	3,778	1,312
Alphabetic ordinality—Dist 1 (Correct)	21.55	3.92	21.07	3.75
Alphabetic ordinality—Dist 2–3 (ms)	4,475	2,077	3,742	1,654
Alphabetic ordinality—Dist 2–3 (Correct)	28.26	8.94	28.36	5.13
Alphabetic ordinality—ordered—Dist 1 (ms)	3,964	1,633	3,601	1,357
Alphabetic ordinality—ordered—Dist 1 (Correct)	10.68	2.79	10.96	2.10
Alphabetic ordinality—ordered—Dist 2–3 (ms)	4,747	2,337	3,789	1,761
Alphabetic ordinality—ordered—Dist 2–3 (Correct)	9.74	4.74	11.96	3.82

HMA = High Math Anxiety; LMA = Low Math Anxiety.

**Figure 2.**
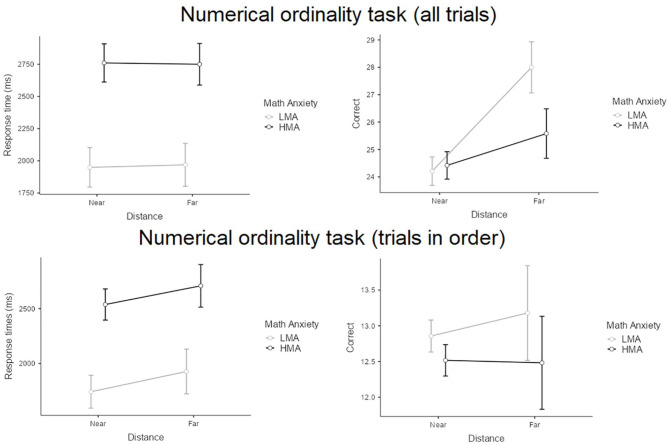
Overview of performance on the numerical ordinality task between the HMA group and the LMA group.

In the numerical ordinal sequencing subtask (see Figure 2), regarding response times across all trials, there was no effect of distance (*p* *=* .933) and no interaction effect (*p* = .795). However, there was a main effect of group, *F*(1,58) = 13.68, *p* < .001, partial η^2^ = .19, indicating that the HMA group was slower than the LMA group. For trials that were arranged in the correct order, we found a significant main effect of distance, *F*(1,58) = 4.65, *p* = .035, partial η^2^ = .08, and a main effect of group, *F*(1,58) = 11.45, *p* *=* .001, partial η^2^ = .17, but no interaction effect. These results indicate that there was an RDE for both groups and that the HMA group performed slower than the LMA group. For the number of correct trials across all trials, we not only found a main effect of distance, *F*(1,58) = 19.38, *p* < .001, partial η^2^ = .25, but also an interaction effect, *F*(1,58) = 5.57, *p* = .023, partial η^2^ = .09. These results indicate that children in the HMA group made fewer correct responses in the far-distance trials. Regarding the number of correct trials for trials in the correct order, there were no main effects of group or interactions (*p*s > .176).

**Figure 3.**
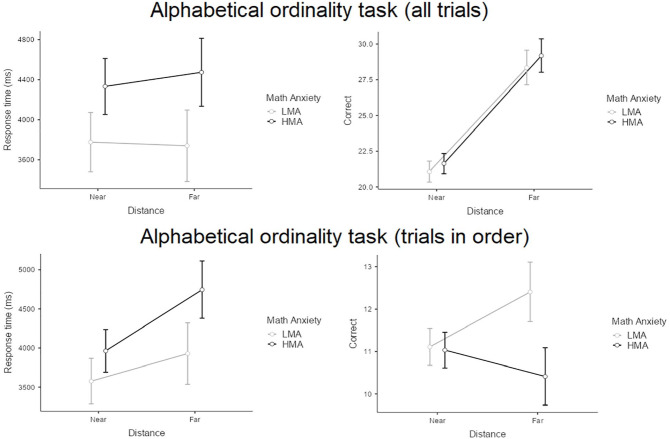
Overview of performance on the alphabetical ordinality task between the HMA group and the LMA group.

For the alphabetical ordinal sequencing task (see Figure 3), regarding response times across all trials, there was no effect of distance (*p* *=* .631), no effect of group (*p* *=* .147), and no interaction effect (*p* = .419). When considering only trials in the correct order, we found a main effect of distance, *F*(1,58) = 8.74, *p* *=* .005, partial η^2^ = .13, but no main effect of group or interaction effect (*p*s > .075). In terms of number of correct responses on all trials, there was a main effect of distance, *F*(1,58) = 81.23, *p* *<* .001, partial η^2^ = .59, but no main effect of group or interaction (*p*s > .712). On correctly ordered trials, there was no effect of distance or main effect of group, but an interaction effect, *F*(1,58) = 6.16, *p* = .016, partial η^2^ = .10, indicating that the HMA group performed worse on far-distance trials.

### What underlies worse performance on ordinality tasks in HMA?

The overall pattern of results presented above indicates that HMA individuals perform worse on both numerical and alphabetical ordinality sequencing tasks. To further enhance our understanding of the performance patterns above, we decided to incorporate covariates in the ANOVAs (i.e., ANCOVAs) to try to further disentangle the mechanisms behind the affected performance in HMA individuals. Given that there are significant correlations between performance on the ordinality tasks and measures of shifting ability and digit comparison performance, we repeated the ANOVAs with those variables included in the models. The poorer performance displayed by the HMA group on the numerical ordinality task could potentially be attributed to poorer overall symbolic number processing, as indexed through the symbolic number comparison task. In addition, performance on the numerical ordinality task could also be affected by EF, in this case, through the shifting component.

Focusing on overall response times on the numerical ordering task, while including response time for the digit comparison task and shifting as covariates, we find that both digit comparison and shifting ability are related to performance on the numerical ordinality task, *F*(1,56) = 20.03, *p* < .001, partial η^2^ = .26 and *F*(1,56) = 4.33, *p* = .042, respectively. After controlling for these variables, we still find a significant effect of group, *F*(1,56) = 8.58, *p* = .005, partial η^2^ = .13. Looking at the number of correct trials, we find that only shifting ability, as a covariate, is significantly related to performance, *F*(1,56) = 10.57, *p* = .002, partial η^2^ = .16. Thus, the effect of group disappears once controlling for shifting. When considering only correctly ordered trials and response times on the numerical ordinality task, we again find that digit comparison performance is related to performance, *F*(1,56) = 16.55, *p* < .001, partial η^2^ = .23 while shifting ability was not *F*(1,56) = 3.81, *p* = .056, partial η^2^ = .07. There was still an effect of group while controlling for these variables *F*(1,56) = 5.65, *p* *=* .021, partial η^2^ = .09. In terms of correctly responded trials for correctly ordered trials, only shifting ability was significantly related to the number of correct responses, *F*(1,56) = 6.35, *p* = .015, partial η^2^ = .10, thereby eliminating the effect of group.

For the alphabetical ordinal sequencing task, there were overall no main effects of group in the ANOVAs reported above. However, given the observed interaction effect, we included the shifting ability as a covariate for follow-up analysis. There was no interaction between shifting ability and the number of correct responses (*p* *=* .063) or group and correct responses (*p* = .078), but shifting ability was significantly related to performance overall, *F*(1,56) = 4.97, *p* = .030, η^2^ = .08.

Taken together, although the HMA group showed fewer correct responses than the LMA group in far-distance trials in the alphabetical ordinality task, this seemed to be attributable to lower shifting ability. Meanwhile, the HMA group’s worst performance on the numerical ordinality task remained while including the aforementioned covariates in the models.

## Discussion

The primary aim of the current study was to achieve a better understanding of how MA potentially interferes with mathematical performance in middle school children. Although researchers have contributed significantly to our understanding of MA in children, there are still unresolved issues regarding whether MA is characterised by less proficient numerical skills or EF. To address these issues, we were particularly interested in looking at those children located at the tail-ends of the distribution of MA scores. The children at the higher end of the distribution could arguably suffer from “high” MA, whereas children at the lower end would be composed of children with “low” or conceptually a nonexistent level of MA. Initial analyses corroborate the widely reported link between MA and math abilities, where the HMA group displayed poorer performance on both arithmetic fluency and calculation skills. Given the extant evidence suggesting that a high level of MA undermines WM resources, in line with the so-called “affective drop” hypothesis of MA (e.g., Ashcraft & Faust, 1994), we hypothesised that children in the HMA group would show lower WM performance than the LMA group. However, to our surprise, we failed to find such an effect, which is a finding that warrants some elaboration. The expectation that children with HMA would show lower WM capacity is premised upon the notion that these children would be most susceptible to interruptions in WM due to stressful responses to mathematics, which in turn disrupts math performance. The link between WM and MA was first established by Ashcraft and Faust (1994) and Faust and colleagues (1996), who found that college students with HMA performed worse on calculation problems that required a carry operation (e.g., 27 + 18), which presumably taxes WM resources. However, it is far from self-evident that it is the size of the mental workspace (e.g., number of items recalled) in WM that is the crucial and contributing factor. In fact, Ashcraft and Kirk (2001) hypothesised that it was the central executive component of WM that contributed to performance deficits in highly math-anxious college students. In the context of MA, poorer central executive processes result in inefficient and slower application of various procedures during arithmetic problem solving (Ashcraft & Kirk, 2001). In light of this reasoning, the similarity in performance in the HMA group and the LMA group on the WM task could potentially be due to the task not eliciting sufficient processing load on the central executive during recall. So, although there were no differences in WM capacity between the groups, the HMA group showed significantly slower performance on the trail-making task, which mainly taps into the shifting component of EF. It could be argued that the shifting component, as measured by our task, places more load on the central executive component, thus showing stronger links with MA. This finding is congenial with Zivkovic et al. (2022), who also found a specific link between shifting, MA, and math performance. This is also in line with neuroimaging data on MA and EF, where shifting was impacted in high-math-anxious university students (Pizzie et al., 2020). Students with HMA displayed lower neural activity in the central executive network, comprised of areas such as the bilateral IPS and lateral prefrontal cortex. Task-evoked engagement of the central executive network is often reported to be accompanied by a deactivation of the default mode network (DMN). Pletzer and colleagues (2015) conducted an fMRI study using two groups of adults with low or high MA. The authors found that MA leads to ineffective deactivation of the DMN. The DMN is engaged in processes, such as self-referencing, emotional processing, and long-term memory processing, when there are no immediate demands on the central executive network that is engaged during goal-directed and effortful processing. Pletzer et al. (2015) hypothesised that this reduced deactivation indicates a preoccupation with the emotional value of the stimuli, leading to a decreased ability to inhibit irrelevant information during mathematical problem solving. Taking into account that the trail-making task used in our study task involves letters as well as numbers as stimuli, it could very well be the case that math-anxious children found it more challenging to effectively allocate central executive resources to perform the shifting task, which would be congruent with the DMN and CEN network dynamics reported by Pletzer et al. (2015) and Pizzie et al. (2020). This reasoning would also support recent findings on attentional control and MA using eye-tracking, where the researchers found that children with MA failed to allocate task-relevant attentional resources while disregarding task-irrelevant information in a math problem solving situation (Li et al., 2023).

The finding that shifting ability was implicated in children with MA also feeds into our central findings about cardinality and ordinality processing. We did not find that children in the HMA group showed any deviant capabilities on tasks involving cardinality processing, neither in the symbolic task nor the non-symbolic task. The link between MA and cardinality processing has mostly been reported in adults (e.g., Dietrich et al., 2015; Douglas & Lefevre, 2017; Georges et al., 2016; Lindskog et al., 2017; Maldonado Mosco et al., 2022; Maloney et al., 2011; Núñez-Peña & Suárez-Pellicioni, 2014). As opposed to cardinality performance, we find aberrant performance in math-anxious children concerning ordinality tasks, most pronounced in the numerical sequencing condition. Overall, we replicated the RDE, but we also found that children in the HMA group performed slower and made more errors on far-distance trials. On the alphabetical sequencing task, we mainly found that children in the HMA group struggled with far-distance trials. This seems to indicate that children with MA have a pronounced difficulty with ordering elements in the numerical domain but also have a more general difficulty in ordering elements in a sequence, regardless of the stimulus domain. Responses on far-distance trials likely involve additional processes above and beyond immediate access to the internal count list for verification, such that additional cognitive load is required for rearranging the elements in the set visible on the screen. It could very well be the case that individuals with great affinity with the count list do not need to engage their EF resources to verify the veracity of the triplets shown, while other individuals with a less internalised mental number line need to recruit EF resources to deduce whether the triplets are in order or not. Our follow-up analyses, with the inclusion of shifting ability as a covariate in our models, suggest that this aspect of EF is a contributing factor to successful performance on ordinality processing—both in numerical ordering and alphabetical ordering. Hence, we corroborate the findings by Colomé and Núñez-Peña (2021), who found impaired ordinality processing in adults with MA. Trying to reconcile the disparate findings in different age groups, a tentative explanation is that EF is indeed tied to math abilities and MA in middle school. Impaired EF in these children leads to less affinity with basic aspects of the number line and has downstream effects on learning and mastering arithmetical principles, with the consequence that these children and adolescents are less inclined to engage with mathematics curricula and subject matter throughout their educational career. This may affect the degree to which they hone their magnitude processing skills, non-symbolically or symbolically, compared with their less math-anxious peers, resulting in poorer performance on basic number processing tasks apparent in studies on young adults.

It is noteworthy that the mean age of the HMA group was higher than the LMA group, which would support the notion that MA levels increase as children grow older. A consequence is that these children have slightly more experience with math and more educational opportunities to hone their numerical skills (e.g., the count list). Still they perform worse on our measures, tapping into cognitive abilities as well as math.

Some limitations are worth mentioning. As mentioned above, it may be the case that the verbal WM task used in our study did not put enough stress on executive processes to find an effect. Also, we did not use a measure of inhibition skills, which is the third and final component of the often-used tripartite model of EF (cf. Diamond, 2013; Miyake & Friedman, 2012), which could have resulted in additional information about specific aspects of EF and their relationship to ordinality processing and MA. Another factor worth considering is that we did not administrate an assessment of state-trait anxiety (STAI) that would capture children’s general anxious tendencies. Thus, we cannot exclude the potential role of general anxiety and dissociate it from the effects of MA. MA has often been linked to poor performance in mathematics, such as in the case of DD. In the current study, we did not screen for learning impairments in math. Based on pure probability, a small proportion of the sample with high MA would likely belong to that population. As evident by our initial analyses, we replicate the finding that children with high MA generally show lower math performance. There is strong evidence for a link between numerical ordinality processing ability and math performance (Lyons et al., 2014), which could partly explain our findings regarding MA and numerical ordinal processing. Still, it would not intuitively explain the performance difference on the non-numerical ordinal processing task. However, future studies would benefit from disentangling the role of EF processing in more strictly controlled samples. For example, Mammarella and colleagues (2015) carefully investigated a sample of children with DD with absent MA and children with MA without DD. This provided valuable insight into specific impairments in verbal vs. visuospatial WM in these respective populations. It is also worth mentioning that we used a neuropsychological test (i.e., the trail-making test) to assess shifting ability. This test involves numbers and letters, and even though the counting sequence and the alphabet are well-rehearsed in middle schoolers, it remains an open question as to whether numerical affinity would affect trail-making performance. Our findings should therefore come with the caveat that they should be complemented with follow-up studies using alternative tests of shifting ability (see Zivkovic et al., 2022, for example).

Although primarily developed to assess MA in younger children, we still find effects using the SEMA questionnaire in older children, thus corroborating the study by Sánchez-Pérez and colleagues (2021). However, we acknowledge that by using this questionnaire, the level of MA across the entire sample may have been underestimated. In terms of age, the relationship between MA and ordinality processing may be restricted to older children, mirroring the finding that ordinality processing is a stronger predictor of mathematics achievement in children aged 10–14 years (Lyons et al., 2014). In addition, we used a quite wide age range to correspond to middle school children, which limits the inferential power to make claims about specific developmental stages.

In sum, our novel contribution to the literature lies in the establishment that math-anxious children do not seem to have issues in cardinality processing specifically. However that MA is linked to poorer ability to order elements efficiently compared with non-anxious peers. Although children with MA seem more affected by numerical stimuli than alphabetical stimuli, we here report that a central factor seems to be the involvement of executive processes during ordinality judgements and that EF may constitute the driving force behind these delays in competence.
